# Association of Glycosylated Hemoglobin A1c Level With Sudden Sensorineural Hearing Loss: A Prospective Study

**DOI:** 10.3389/fendo.2021.763021

**Published:** 2021-11-18

**Authors:** Ying Shen, Zhong Zheng, Lili Xiao, Chengqi Liu, Jingyi Guo, Zhengnong Chen, Yaqin Wu, Haibo Shi, Zhen Zhang, Di Qian, Yanmei Feng, Shankai Yin

**Affiliations:** ^1^ Department of Otolaryngology Head and Neck Surgery, Shanghai Jiao Tong University Affiliated Sixth People’s Hospital, Shanghai, China; ^2^ Shanghai Key Laboratory of Sleep Disordered Breathing, Shanghai, China; ^3^ Clinical Research Center, Shanghai Jiao Tong University Affiliated Sixth People’s Hospital, Shanghai, China; ^4^ Department of Otolaryngology, People’s Hospital of Longhua, Shenzhen, China

**Keywords:** sudden sensorineural hearing loss, diabetes mellitus, glycosylated hemoglobin A1c, microvascular disorders, propensity score matching

## Abstract

Glycosylated hemoglobin A1c (HbA1c) level has strong relevance to microvascular disorders, which are also thought to be the current main aspect of sudden sensorineural hearing loss (SSNHL), so we aim to elucidate the association of the HbA1c level with the severity, types, and prognosis of SSNHL. In this study, comparative analyses based on propensity score matching of the severity, types, and prognosis of SSNHL with the HbA1c level in 116 patients diagnosed as SSNHL were conducted, where they were divided into diabetes mellitus (DM) group and non-DM group. We finally found that, among patients with SSNHL, diabetic patients had a higher HbA1c level, more severe hearing loss, and poorer prognosis than non-diabetic patients. The HbA1c level was found to be significantly correlated with the severity and types of SSNHL, while no strong relevance was found between the higher HbA1c level and the poorer prognosis of SSNHL.

## Introduction

Sudden sensorineural hearing loss (SSNHL) is generally defined as a hearing loss that is sensorineural in nature. It occurs within a 72-h window and consists of a decrease in hearing of greater than 30 dB, affecting at least three consecutive frequencies (1). It has a prevalence of five to 27 individuals per 100,000 people in the United States (2). The main pathogenic causes of SSNHL are still unidentified, yet several pathophysiological mechanisms have been proposed, including viral infection, vascular disorders, autoimmune disease, and other causes, such as cochlear membrane rupture (3).

Among the various possible etiologies and pathogenesis, mainstream pathophysiological mechanisms for SSNHL attribute its occurrence to microvascular disorders. Microvascular damage as well as other microcirculatory disturbances involving sudden increases in blood viscosity, along with embolic and thrombotic episodes (4) such as within the labyrinthine artery, can interrupt the vascular supply to the cochlea, eventually causing cochlea dysfunction and resulting in SSNHL.

Diabetes mellitus (DM) contributes the greatest risks to microvascular disorders. Diabetic kidney diseases, diabetic retinopathy, and diabetic foot are a few typical long-term microvascular complications of DM patients resulting from extended poorly controlled high blood sugar levels. Accounting for 90–95% of all DM, type 2 diabetes mellitus (T2DM) frequently goes undiagnosed for many years as hyperglycemia develops gradually. The American Diabetes Association states that even undiagnosed T2DM patients are at an increased risk of developing both macrovascular and microvascular complications (5).

The glycosylated hemoglobin A1c (HbA1c) level has been widely recognized as a reliable diagnostic biomarker for DM, especially for T2DM. About 6% of hemoglobin A can be categorized as glycated hemoglobin (6) and, among those, HbA1c is composed of approximately 5% glycated hemoglobin in a healthy individual (7). It forms when glucose attaches specifically to the N-terminal valine of the β subunit of hemoglobin and reflects the average serum glucose concentration over the lifespan of an erythrocyte, which is on average 120 days (8). Thus, HbA1c testing is helpful for clinicians to assess the long-term glucose control status of the patient. A previous study has shown that, for every 1% rise in HbA1c level, there is a 37% increase in the risk of a microvascular disease (9). Although many studies have highlighted the negative role of DM in the pathogenesis and prognosis of SSNHL, few studies have explored the relationship between HbA1c and SSNHL. The present study is designed to elucidate the association of the HbA1c level with the severity, types, and prognosis of SSNHL in a group of well-defined patients.

## Materials and Methods

### Study Population

We performed a prospective study of patients admitted to the Department of Otolaryngology Head and Neck Surgery at Shanghai Jiao Tong University Affiliated Sixth People’s Hospital, Shanghai, China, with a diagnosis of unilateral SSNHL from August 1, 2018 through December 31, 2020. All patients met the criteria: sensorineural in nature, occurred within a 72-h window, and decrease in hearing of greater than 30 dB affecting at least three consecutive frequencies; unilateral onset; age of 18 years or older; examined less than 3 weeks after the onset of disease; and not receiving a previous therapy. Patients with any accompanying disease or trauma that contributes to hearing loss, such as Meniere’s disease, vestibular schwannoma, or other central nervous system disease, head injury, or use of ototoxic medicine were excluded. Eventually, a total of 116 patients were enrolled in our research. The diagnostic criteria for DM were based on the report of the expert committee on the diagnosis and classification of diabetes mellitus (10), and the participants were divided into two groups with SSNHL: the DM group and the non-DM group according to their previous medical history and blood tests for DM.

### Measurements and Treatment Process

All patients underwent general clinical interviews, laboratory testing, otoscopic examinations, complete audiologic tests, including pure-tone audiometry, tympanogram tests, and otoacoustic emission measures. Internal auditory canal magnetic resonance imaging was also conducted to exclude retrocochlear pathology, and each patient was provided with personal protection devices such as ear cover and earplug during the inspection.

Auditory evaluations were mainly determined by pure-tone average (PTA) from 125 Hz to 8 kHz through measuring air and bone conduction, which were all performed by certified and experienced audiologists. Pure-tone audiometry was conducted in sound-proofed rooms with noise less than 30 dB(A) using a calibrated GSI-61TM audiometer (United States) coupled with TDH 39 headphones. The hearing thresholds of all SSNHL patients were expressed as the mean hearing level of impaired frequencies. According to the report of the First Informal Consultation on Future Programme Developments for WHO-PDH, 1997 (11), hearing was defined as normal if PTA was less than 25 dB HL, and the severity of hearing loss was defined as “mild” if PTA was 26 dB HL but less than 40 dB HL, “moderate” if it was greater than 41 dB HL but less than 60 dB HL, “severe” if it was greater than 61 dB HL but less than 80 dB HL, and “profound” if it was greater than 81 dB HL. Furthermore, we determined the different types of SSNHL according to the Guidelines for Diagnosis and Treatment of Sudden Deafness, 2015, China (12), which are judged by the audiometric configurations of patients, including their impaired frequencies and degree of hearing loss. It was classified as “low-frequency descending type of SSNHL (LF-SSNHL)” if hearing loss happened at frequencies below and including 1 kHz, “high-frequency descending type of SSNHL (HF-SSNHL)” if hearing loss happened at frequencies above and including 2 kHz, “flat type of SSNHL (FT-SSNHL)” if hearing loss happened at all frequencies and the mean hearing level was less than or equal to 80 dB HL, and “total deafness of SSNHL (TD-SSNHL)” if the mean hearing level was greater than or equal to 81 dB HL.

For laboratory testing, all patients had fast blood samples taken in the morning after 8 h of fasting, which included fasting plasma glucose (mmol/L), glycosylated hemoglobin A1c (HbA1c, %), serum creatinine (μmol/L), D-D dimer (mg/L fibrinogen equivalent unit), total cholesterol (mmol/L), triglycerides (mmol/L), high-density lipoprotein (mmol/L), and low-density lipoprotein (mmol/L). Thereafter, the HbA1c test was performed using a certified method by the NGSP (www.ngsp.org), reported in %HbA1c. The analyses of all blood samples were performed by routine laboratory screening in our hospital central laboratory.

After admission, all patients were treated with comprehensive treatments, including the use of systemic steroids with initial doses of methylprednisolone at 80 mg intravenously, oral antioxidants, and neurotrophic drugs. Patients with “FT-SSNHL” and “TD-SSNHL” took an additional drug of batroxobin which lowers fibrinogen, with an initial dose of 10 BU and then 5 BU for subsequent administrations according to the Guidelines for Diagnosis and Treatment of Sudden Deafness, 2015, China (12). The serum fibrinogen level was monitored before each use to confirm it is higher than 1 g/L to avoid bleeding and other related side effects. During the treatments, blood glucose was monitored regularly through fixed-point monitoring of blood glucose from fingerstick testing for five times a day that covered timepoints of after fasting, 2 h after three meals, and at 21 PM. When the blood glucose of a patient fluctuated, an endocrinologist would be consulted in time and modify his hypoglycemic treatments. Furthermore, patients whose blood glucose fluctuates greatly or was difficult to control had their systemic steroids therapy dosage modified or even stopped immediately. It should be stressed that we defined treatment course here as the duration of systemic steroids. All SSNHL patients would receive treatments of antioxidants and neurotrophic agents for 2 weeks.

We were able to obtain follow-up auditory evaluations for each patient on day 14 after treatments. The prognosis of SSNHL was judged by the post-treatment hearing thresholds of PTA in impaired frequencies. Treatment was deemed effective if the PTA of the impaired frequencies improved by at least 15 dB or when the damaged ear achieved the same level as the normal or unaffected ear of the patient. It was deemed ineffective in the treatment of SSNHL if the PTA of the impaired frequencies improved by less than 15 dB (12).

### Statistical Analysis

Categorical variables were expressed as number and percentage. Data were investigated using Kolmogorov–Smirnov test to determine the distribution pattern. The data of clinical character did not have a normal distribution (*P* < 0.05); thus, quantitative variables were presented as median (inter-quartile range, IQR). We adopted a non-parametric test (Mann–Whitney *U*-test) for quantitative variables and *χ* ^2^ test or Fisher’s exact test for categorical variables. Correlations between HbA1c level and variables were sought using the Spearman rank correlation test. Univariate analysis was carried out to assess differences in demographic, clinical, and metabolic variables, accompanying symptoms, severity of hearing loss, and types of SSNHL between diabetic and non-diabetic groups. To overcome selection biases in this research, we performed a 1:1 match for each patient by using propensity score matching (PSM). A two-sided *p*-value <0.05 was taken to indicate statistical significance. All statistical analyses were performed with the aid of SPSS version 26 (SPSS, USA).

## Results

### Clinical Characteristics of Patients Before PSM

Of the 116 SSNHL patients enrolled (58 men [50.0%]; 58 women [50.0%]; median (IQR) age, 54.00 [25.25] years), 31 patients had DM (DM group) and 85 were non-diabetic (non-DM group). The course of DM in these 31 diabetic patients was 10.89 [6.89] years on average. The clinical characteristics of the study populations are presented in **Table 1**. There were no statistically significant differences in gender, time to treatment initiation, the side of the affected ear, tinnitus and vertigo associated incidence, admission systolic blood pressure, admission diastolic blood pressure, serum creatinine, D-D dimer, total cholesterol, triglycerides, high-density lipoprotein and low density lipoprotein between the two groups (all P > 0.05). We noted that diabetic patients were much older than non-diabetic ones (61.00 [15.00] vs 49.00 [26.00] years, P<0.05). Meanwhile, the FBG (10.85 [6.89] vs 6.19 [1.91] mmol/L) and HbA1c level (7.30 [1.50] vs 5.60 [0.40] %) (both P<0.05) were higher in the diabetic patients. However, it was worth mentioning that steroid medications had glycemic effects and thus FBG might been greatly affected by using steroid medications in a short time. We did not included FBG in the subsequent analysis.

**Table 1:** Clinical characteristics of study participants before propensity score matching.

Indicators	DM group (*n* = 31)	Non-DM group (*n* = 85)	*P*-value
**Demographics**			
Gender (male/female)	16:15	42:43	0.834
Age (years)	61.00 (15.00)	49.00 (26.00)	<0.001*
Time to treatment initiation (days)	5.00 (5.00)	4.00 (5.00)	0.616
Side (left/right)	15:16	43:42	0.834
**Clinical and metabolic variables**			
SBP (mmHg)	129.00 (22.00)	122.00 (15.00)	0.067
DBP (mmHg)	76.00 (15.00)	78.00 (14.00)	0.726
BMI (kg/m^2^)	22.72 (5.62)	23.42 (4.00)	0.925
FBG (mmol/L)	10.85 (6.89)	6.19 (1.91)	<0.001*
HbA1c (%)	7.30 (1.50)	5.60 (0.40)	<0.001*
Scr (umol/L)	59.00 (21.50)	59.00 (18.00)	0.849
DD (mg/L FEU)	0.16 (0.15)	0.17 (0.13)	0.886
TC (mmol/L)	5.20 (0.82)	5.11 (1.31)	0.757
TG (mmol/L)	0.99 (0.54)	0.90 (0.94)	0.107
HDL (mmol/L)	1.36 (0.37)	1.38 (0.53)	0.135
LDL (mmol/L)	3.05 (1.05)	3.01 (1.19)	0.898
Pre-treatment PTA of the impaired frequencies (dBHL)	91.67 (19.17)	66.67 (37.38)	<0.001*
Treatment course (days)	5.00 (2.50)	5.00 (0)	0.011*
Post-treatment PTA of the impaired frequencies (dBHL)	80.83 (35.00)	42.86 (43.72)	<0.001*
**Effective case (*n*, %)**	**10 (32.3%)**	**50 (58.8%)**	0.011*
**Accompanying symptoms (*n*, %)**			
Vertigo	17 (56.7%)	36 (42.4%)	0.232
Tinnitus	26 (86.7%)	72 (84.7%)	0.912
**Severity of hearing loss (*n*, %)**			
Mild to moderate	3 (9.7%)	37 (43.5%)	0.001*
Severe to profound	28 (90.3%)	48 (56.5%)	
**Audiometric configurations (*n*, %)**			
LF-SSNHL	1 (3.2%)	10 (11.8%)	<0.001*
HF-SSNHL	2 (6.5%)	4 (4.7%)	
FT-SSNHL	4 (12.9%)	44 (51.8%)	
TD-SSNHL	24 (77.4%)	27 (31.8%)	

DM, diabetes mellitus; SBP, admission systolic blood pressure; DBP, admission diastolic blood pressure; BMI, body mass index; FBG, fasting plasma glucose; HbA1c, glycosylated hemoglobin A1c; Scr, serum creatinine; DD, D-D dimer; TC, total cholesterol; TG, triglycerides; HDL, high-density lipoprotein; LDL, low-density lipoprotein; PTA, pure-tone average; SSNHL, sudden sensorineural hearing loss; LF-SSNHL, low-frequency descending type of SSNHL; HF-SSNHL, high-frequency descending type of SSNHL; FT-SSNHL, flat type of SSNHL; TD-SSNHL, total deafness of SSNHL.

*p < 0.05 (statistically significant). The bold values in “Effective cases (n,%)” were the number or percentage of SSNHL patients who were classified as effective according to the outcome evaluation criteria. The bold values in “P values” mean the statistical indicators that were taken as significant when smaller than 0.05.

Besides, diabetic patients’ pre-treatment PTA of the impaired frequencies was higher than non-diabetic patients (91.67 [19.17] vs 66.67 [37.38] dBHL, P<0.05) and among all types of SSNHL, “total deafness type of SSNHL” dominated. We further compared the degree of the impaired frequencies’ hearing loss between the two groups and found that the differences in the proportion of “mild to moderate” and “severe to profound” hearing loss were statistically significant (P< 0.05) (as shown in **Table 1**). Meanwhile, the treatment course for patients with DM was also shorter than those without DM (P<0.05), mainly due to worse blood glucose control in diabetic SSNHL patients.

While focusing on the prognosis of SSNHL, we found that post-treatment PTA of the impaired frequencies was much higher in DM group (80.83 [35.00] vs 42.86 [43.72] dBHL, P<0.05). The number of effective cases of non-DM group was also significantly higher than that of DM group (P<0.05). Only 32.3% diabetic SSNHL patients in our research performed effectively in their treatment (as shown in **Table 1**).

### Pathogenic Features in the Matched Data Set After PSM Using “Age” as the Predictor

Considering the significant difference in age, we performed PSM and used “age” as the predictor here to correct for potential bias and further explore the pathogenic features between the two groups, mainly focusing on their level of HbA1c, the severity of hearing loss, and the characteristics of their audiometric configuration. After PSM, 26 pairs of patients were successfully matched (**Table 2**). It was worth noting that the HbA1c level in the DM group was still significantly higher than that in the non-DM group after PSM [7.40 (2.13) *vs.* 5.70 (0.60) %, *P* < 0.05). The diabetic SSNHL patients performed much more poorly in pre-treatment PTA of the impaired frequencies than non-diabetic SSNHL patients [93.08 (19.17) *vs.* 76.43 (35.37) dBHL, *P* < 0.05]. The degree of hearing loss of the impaired frequencies and the audiometric configurations of SSNHL between the two groups also show significant differences (*P* < 0.05). Specifically, “FT-SSNHL” dominated in SSNHL patients without DM, while “TD-SSNHL” was the most common type in diabetic SSNHL patients (as shown in **Table 2**).

**Table 2:** Pathogenic features in the matched data set after propensity score matching using “age” as the predictor.

Indicators	DM group (*n* = 26)	Non-DM group (*n* = 26)	*P*-value
**Demographics**			
Gender (male/female)	16:10	15:11	0.777
Age (years)	59.50 (11.50)	59.00 (11.50)	0.934
Time to treatment initiation (days)	5.00 (5.00)	3.00 (3.75)	0.293
Side (left/right)	13:13	9:17	0.262
**Clinical and metabolic variables**			
SBP (mmHg)	125.00 (26.75)	124.00 (20.25)	0.674
DBP (mmHg)	75.50 (15.00)	77.00 (13.00)	0.510
BMI (kg/m^2^)	22.79 (5.25)	24.49 (3.47)	0.337
HbA1c (%)	7.40 (2.13)	5.70 (0.60)	<0.001*
Scr (umol/L)	58.50 (21.25)	62.50 (15.00)	0.252
DD (mg/L FEU)	0.16 (0.13)	0.19 (0.12)	0.576
TC (mmol/L)	5.18 (0.84)	4.94 (1.48)	0.516
TG (mmol/L)	1.04 (0.48)	1.14 (0.82)	0.534
HDL (mmol/L)	1.34 (0.29)	1.34 (0.37)	0.756
LDL (mmol/L)	3.03 (1.14)	2.78 (1.09)	0.728
Pre-treatment PTA of the impaired frequencies (dBHL)	93.08 (19.17)	76.43 (35.37)	0.011*
**Accompanying symptoms (*n*, %)**			
Vertigo	14 (53.8%)	12 (46.2%)	0.579
Tinnitus	23 (88.5%)	20 (76.9%)	0.463
**Severity of hearing loss (*n*, %)**			
Mild to moderate	2 (7.7%)	8 (30.8%)	0.035*
Severe to profound	24 (92.3%)	18 (69.2%)	
**Audiometric configurations (*n*, %)**			
LF-SSNHL	0	3 (11.5%)	0.042*
HF-SSNHL	2 (7.7%)	2 (7.7%)	
FT-SSNHL	4 (15.4%)	10 (38.5%)	
TD-SSNHL	20 (76.9%)	11 (42.3%)	

*p ＜ 0.05 (statistically significant). The bold values in “Effective cases (n,%)” were the number or percentage of SSNHL patients who were classified as effective according to the outcome evaluation criteria. The bold values in “P values” mean the statistical indicators that were taken as significant when smaller than 0.05.

DM, diabetes mellitus; SBP, admission systolic blood pressure; DBP, admission diastolic blood pressure; BMI, body mass index; HbA1c, glycosylated hemoglobin A1c; Scr, serum creatinine; DD, D-D dimer; TC, total cholesterol; TG, triglycerides; HDL, high-density lipoprotein; LDL, low-density lipoprotein; PTA, pure-tone average; SSNHL, sudden sensorineural hearing loss; LF-SSNHL, low-frequency descending type of SSNHL; HF-SSNHL, high-frequency descending type of SSNHL; FT-SSNHL, flat type of SSNHL; TD-SSNHL, total deafness of SSNHL.

### Prognosis Conditions of SSNHL in the Matched Data Set After PSM Using Prognosis-Related Factors of SSNHL as the Predictors

The prognosis for SSNHL recovery had been reported to be dependent on a number of factors, including the age of patients, presence of vertigo at onset, degree of hearing loss, audiometric configurations, treatment course, and time between onset of hearing loss and treatment (13–15). To remove biases, we performed another 1:1 match for each patient by using PSM where we used prognosis-related factors, including “age”, “treatment course”, “severity of hearing loss”, and “audiometric configurations” as the predictors. Here 19 pairs of patients were eventually matched (as shown in **Table 3**). There were no statistically significant differences in age, course of sudden deafness, vertigo-associated incidence, pre-treatment PTA of the impaired frequencies, treatment course, severity of hearing loss, and different audiometric configurations of SSNHL between the two groups (all *P >*0.05). The result showed that diabetic SSNHL patients had a higher level of HbA1c and much worse performance in post-treatment PTA of the impaired frequencies and the number of effective cases, which represented a poor prognosis of SSNHL. The differences were statistically significant (both *P <*0.05) (as shown in **Table 3**).

**Table 3:** Prognosis conditions of SSNHL in the matched data set after propensity score matching using prognosis-related factors of SSNHL as the predictors.

Indicators	DM group (*n* = 19)	Non-DM group (*n* = 19)	*P*-value
**Demographic, clinical, and metabolic variables**			
Age (years)	56.00 (11.00)	65.00 (15.00)	0.389
Time to treatment initiation (days)	5.00 (5.00)	3.00 (6.00)	0.906
HbA1c (%)	7.40 (2.65)	5.80 (0.40)	<0.001*
Pre-treatment PTA of the impaired frequencies (dBHL)	86.43 (24.17)	81.43 (37.35)	0.139
Treatment course (days)	6.08 (18.77)	5.00 (1.50)	0.472
Post-treatment PTA of the impaired frequencies (dBHL)	75.83 (36.43)	65.00 (40.10)	0.032*
Effective case (*n*, %)	5 (26.3%)	11 (57.9%)	0.049*
**Accompanying symptoms (*n*, %)**			
Vertigo	10 (52.6%)	8 (42.1%)	0.516
**Severity of hearing loss (*n*, %)**			
Mild to moderate	3 (15.8%)	6 (31.6%)	0.447
Severe to profound	16 (84.2%)	13 (68.4%)	
**Audiometric configurations (*n*, %)**			
LF-SSNHL	1 (5.3%)	1 (5.3%)	0.703
HF-SSNHL	2 (10.5%)	2 (10.5%)	
FT-SSNHL	3 (15.8%)	6 (31.6%)	
TD-SSNHL	13 (68.4%)	10 (52.6%)	

DM, diabetes mellitus; HbA1c, glycosylated hemoglobin A1c; PTA, pure-tone average; SSNHL, sudden sensorineural hearing loss; LF-SSNHL, low-frequency descending type of SSNHL; HF-SSNHL, high-frequency descending type of SSNHL; FT-SSNHL, flat type of SSNHL; TD-SSNHL, total deafness of SSNHL.

*p ＜ 0.05 (statistically significant). The bold values in “Effective cases (n,%)” were the number or percentage of SSNHL patients who were classified as effective according to the outcome evaluation criteria. The bold values in “P values” mean the statistical indicators that were taken as significant when smaller than 0.05.

### Correlation Between HbA1c Level and SSNHL

The level of HbA1c was closely correlated with impaired hearing thresholds, the severity of hearing loss, and different audiometric configurations of SSNHL after PSM (all *P* < 0.05) (as shown in **Table 4**). Since the Spearman correlation coefficients of these three parameters were all positive, the result revealed that the higher level of HbA1c in the patients with SSNHL translates to more severe hearing loss as reflected by “TD-SSNHL” dominating the different audiometric configurations of SSNHL, including “LF-SSNHL”, “HF-SSNHL”, and “FT-SSNHL” (as shown in **Table 4**). Nevertheless, the prognosis of SSNHL had no significant correlation with HbA1c, neither with their post-treatment PTA of the impaired frequencies nor with the number of effective cases (both *P >*0.05) (as shown in **Table 4**).

**Table 4:** Correlation between the HbA1c level and SSNHL.

Parameters	Spearman *r*	*P*-value
Pre-treatment PTA of the impaired frequencies^a^	0.423	0.002*
Severity of hearing loss^a^	0.421	0.002*
Audiometric configurations^a^	0.336	0.015*
Post-treatment PTA of the impaired frequencies^b^	0.318	0.052
Effective case^b^	-0.075	0.652

PTA, pure-tone average.

a We performed a 1:1 match for each patient by using propensity score matching (PSM), choosing “age” as the predictor here.

b We performed a 1:1 match for each patient by using PSM, choosing prognosis-related factors of SSNHL as the predictors here.

*p ＜ 0.05 (statistically significant). The bold values in “Effective cases (n,%)” were the number or percentage of SSNHL patients who were classified as effective according to the outcome evaluation criteria. The bold values in “P values” mean the statistical indicators that were taken as significant when smaller than 0.05.

## Discussion

SSNHL is a sudden illness causing a decrease in hearing of greater than 30 dB that affects at least three consecutive frequencies within a 72-h window. Although it effects a wide population, the main pathogenic causes remain unidentified. Among various possible etiologies and pathogenesis, microvascular disorders are the current main suspect linked to the pathophysiological mechanisms proposed for SSNHL. Microangiopathy, which is common in DM patients, happens in multiple organ systems and could also affect the inner ear vessels like the labyrinthine artery. As an artery without collateral compensation, the labyrinthine artery supplies the cochlea, a highly metabolic organ that is extremely sensitive to ischemia and hypoxia (16, 17). When chronic or transient microvascular disorders happen in the inner ear, a series of pathophysiological changes would lead to cochlear microcirculation dysfunction and eventually cause hearing loss.

Several histological changes have been observed in preceding related studies that support such hypothesis. In the inner ears of diabetic experimental models or patients, thickening of the basement membrane of the capillaries within the stria vascularis on the lateral walls of the cochlea and narrowing of the internal auditory artery had been found (18–20). Other aspects of the cochlea, such as the loss of Corti organs and spiral ganglion neurons and demyelination of the auditory nerve, had also been confirmed in other studies (21, 22).

From clinical aspects, studies have clearly demonstrated DM to be one of the risk factors for hearing loss, including SSNHL. A large-sample cohort study in South Korea, after adjusting for multiple potential confounding factors, including demographic characteristics, occupational noise exposure, lifestyle risk factors, and other metabolic abnormalities, found a strong correlation in risk of hearing loss and DM (23). In terms of SSNHL, a recent study that recruited 26,556 patients with DM showed a significant association between DM and an increased risk of developing SSNHL. Meanwhile, such risk was proportional to the severity of DM (24). Another present study demonstrated that the severity of hearing loss in patients with SSNHL was influenced by the presence of DM. A subgroup of SSNHL patients with T2DM was associated with more severe hearing loss (25). In addition, DM was also found to be one of the adverse factors for SSNHL prognosis (26).

Given the abovementioned data, diabetic patients are more likely to suffer sudden deafness and higher severity in hearing loss level and tend to show a poorer prognosis. Furthermore, some research also found that the risk of hearing loss increased progressively with increasing HbA1c levels above 5%, suggesting that glucose control may play a role in the development of hearing loss in diabetic patients (23). HbA1c, due to the stability of its Amadori form, is clinically recognized as a better reflection of the plasma glycemic status over the past 2 to 3 months (27). For every 1% rise in HbA1c, there is a 37% increase in microvascular diseases (9). Much work has been done to explore the relationship between DM and SSNHL, especially focusing on its microvascular disorders. Until now, few studies to date have analyzed the association between HbA1c and SSNHL.

In this study, we elucidated the association of the HbA1c level with the severity, types, and prognosis of SSNHL in a group of well-defined patients. The clinical and audiometric differences between diabetic and non-diabetic patients with SSNHL were also demonstrated. Among the patients with SSNHL in the present study after PSM, those who met the diagnostic criteria for DM had a higher level of HbA1c and were more severe in their pre-treatment PTA of the impaired frequencies. The proportion of degrees of the hearing loss of the impaired frequencies and audiometric configurations of SSNHL was statistically significant between the two groups. “TD-SSNHL” was dominantly present in SSNHL patients with DM. All these findings indicated that SSNHL patients with DM performed worse in hearing loss at the beginning of SSNHL. The SSNHL prognosis was also worse for diabetic patients. After PSM, more patients in the DM group had a higher post-treatment PTA of the impaired frequencies, and the number of effective cases was less than that in the non-DM group. These results were quite consistent with previous researches.

Based on these findings, we further explored the correlation between the HbA1c level with pre-treatment PTA of the impaired frequencies, severity of hearing loss, different audiometric configurations, and prognosis of SSNHL. We found that the HbA1c level correlated significantly with the first three parameters, which meant that the higher level of HbA1c in the patients with SSNHL, the more serious is the hearing loss in the impaired frequencies pre-treatment, and among all audiometric configurations of SSNHL, “TD-SSNHL” dominated. This revealed a potential connection between SSNHL and glycemic status, suggesting that long-term unsatisfactory blood sugar control may be related to the worse performance in the severity of SSNHL. Considering that a rise in HbA1c meant an increase in microvascular diseases (9), our findings might indirectly support the microvascular etiology hypothesis of SSNHL. It also reminded us that we need to pay close attention to the glucose levels in SSNHL patients and take active measures to control the blood sugar. Given that we found no strong association between the HbA1c level and a prognosis of SSNHL, long-term uncontrolled blood sugar may play a more dominant role in the onset of SSNHL than its progression. The exact mechanism behind it has still not yet been reported. Further work is needed.

There were a few limitations which could be potential sources of bias in this research, including a small sample size limited to a single medical center and measurements of limited parameters. Larger studies containing real-world evidence or multicenter database are required. In addition, the HbA1c level only represents the long-term blood sugar control (about 2 to 3 months) of the subjects. Whether the immediate and/or short-term level of blood glucose is also related even more sensitively is still unknown. To remedy this, for subsequent studies, we plan to take additional parameters like taking the capillary blood glucose concentrations of patients from fingerstick testing before any treatments and the fasting plasma glucose and glycated albumin level to fully reflect the blood glucose levels of the patients. We will also consider enroling diabetic patients who have not yet developed SSNHL to explore whether blood sugar control is also related to the onset of sudden deafness. A lack of follow-up auditory evaluations was also one of the deficiencies of this study. In the future, we hope to conduct more comprehensive assessments of long-term auditory evaluations to reflect the final hearing condition of patients more accurately.

## Conclusions

Among patients with SSNHL, diabetic patients had a higher HbA1c level, more severe hearing loss, and poorer prognosis than patients who were without any history of DM. Furthermore, the HbA1c level significantly correlated with the severity of SSNHL, which revealed that the higher the level of HbA1c in the patients with SSNHL, the more serious the hearing loss in the impaired frequencies and among all audiometric configurations of SSNHL, dominated by “TD-SSNHL”. No strong correlation between the HbA1c level and the prognosis of SSNHL was found in our research. Further studies are needed to determine the molecular mechanisms of hyperglycemia and its related microvascular disorders acting on SSNHL.

## Data Availability Statement

The original contributions presented in the study are included in the article/supplementary material. Further inquiries can be directed to the corresponding authors.

## Ethics Statement

The study protocol was approved and implemented according to the ethical standards of the Shanghai Jiao Tong University Affiliated Sixth People’s Hospital ethics committee [2018-KY-036 (K)]. The trial registration number is ChiCTR18000170072. Written informed consent was obtained before participation. The information of the patients was anonymized and de-identified prior to analysis. The progress was conducted in accordance with the spirit of the Helsinki Declaration.

## Author Contributions

YF and ZheZ have given substantial contributions to the conception and the design of there search. YS and ZhoZ contributed to the acquisition, analysis, and interpretation of the data. All authors have participated in drafting the manuscript. YF, ZheZ and DQ revised it critically. All authors contributed to the article and approved the submitted version.

## Funding

This research was funded by a grant from International Cooperation and Exchange of the National Natural Science Foundation of China (NSFC; 81720108010) to SY and the National Natural Science Foundation of China (no. 81771015) to YF.

## Conflict of Interest

The authors declare that the research was conducted in the absence of any commercial or financial relationships that could be construed as a potential conflict of interest.

## Publisher’s Note

All claims expressed in this article are solely those of the authors and do not necessarily represent those of their affiliated organizations, or those of the publisher, the editors and the reviewers. Any product that may be evaluated in this article, or claim that may be made by its manufacturer, is not guaranteed or endorsed by the publisher.
